# Myocardial infarction in a pregnant woman revealing a transitional deficit in protein S: a rare case report

**DOI:** 10.11604/pamj.2019.34.27.18614

**Published:** 2019-09-12

**Authors:** Souleymane Diakite, Fatima Zohra Radi, Jamila Zarzur, Mohamed Cherti

**Affiliations:** 1 Departement of Cardiology B, CHU Ibn Sina, Mohamed V University Rabat, Rabat, Morocco

**Keywords:** Myocardial infarction, pregnancy, protein S deficiency

## Abstract

The occurrence of myocardial ischemia and myocardial infarction in pregnancy is relatively rare, the occurrence of myocardial infarction in pregnancy is associated with cardiovascular risk factors. The deficiency of coagulation regulatory systems in the occurrence of venous thrombosis is well established; however, their role in arterial thrombosis is controversial. Here, we present an interesting case of a 34-year-old of acute myocardial syndrome without persistent ST segment elevation, which revealed transient protein S deficiency. Management of acute coronary syndrome (ACS) during pregnancy may represent a unique clinical challenge. In this manuscript, we review the clinical presentation, anatomic considerations, and management strategy in our patient presenting with ACS. Objective: this case highlights the importance of multimodality approach to help to obtain a more timely diagnosis of myocardial infarction in pregnancy and the importance collaboration between obstetricians, cardiologists, pediatricians and anesthesiologists to ensure optimal care.

## Introduction

The occurrence of myocardial ischemia and myocardial infarction (MI) in pregnancy is relatively rare compared to other complications described in various reports on maternal morbidity and mortality. The occurrence of myocardial infarction in pregnancy is associated with cardiovascular risk factors such as arterial hypertension, diabetes, thrombophilia, preeclampsia and a history of myocardial ischemia [1]. The deficiency of coagulation regulatory systems in the occurrence of venous thrombosis is well established; however, their role in arterial thrombosis is controversial [2]. We describe a case of acute myocardial syndrome without persistent ST segment elevation in the per-partum period in a young woman with transient protein S deficiency. We will review the literature for the risk factors related to pregnancy of myocardial infarction.

## Patient and observation

**Figure 1:**
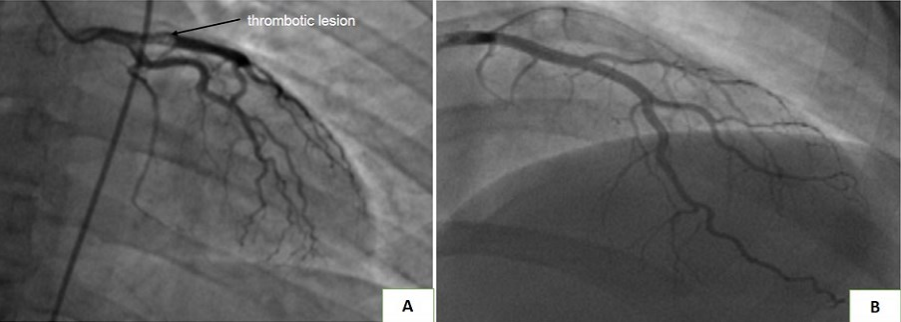
A) demonstrated the presence of a thrombotic lesion on the proximal segment of the left anterior descending (LAD), with a TIMI 3 flow; B) control coronarography performed radially showed the complete disappearance of the thrombotic lesion

A 34 years old female, pregnant of 35 weeks of amenorrhea, was admitted to Cardiology Department because of an inaugural large and atypical chest pain. His past medical history revealed no previous hospitalizations and no cardiovascular risk factors. The clinical examination was without abnormalities and the electrocardiogram (ECG) performed at admission, showed sub-epicardial ischemia current apico-lateral. Laboratory studies have shown a troponin at 125ng/ml (VN < 0.1ng/ml), followed by downward kinetics (102ng/ml). Echocardiography demonstrated akinesia of the antero-septal (AS) and infero-septal walls with systolic dysfunction LV (FESLV = 45% Simson biplane) and alteration of global longitudinal Strain (16%). The patient underwent emergency coronary angiography, which demonstrated the presence of a thrombotic lesion on the proximal segment of the left anterior descending (LAD), with a TIMI 3 flow (Figure 1A), no invasive intervention was undertaken. The patient managed on aspirin, IV unfractionated heparin, and clopidogrel while in hospital, and was discharged on aspirin, clopidogrel and heparin and has done well throughout follow-up. Given the young age, the pregnancy site and the occurrence of myocardial infarction, a thrombophilia assessment was performed (Table 1). Since obstetric conditions being favorable, a natural delivery was allowed, two weeks after the patient gave birth to a newborn baby of 3100g female with apgar scores at 10/10 minutes of life. After 48 hours of delivery, the patient was transferred to the cardiology department to be monitored in a simple follow-up mode. A control coronarography performed showed the complete disappearance of the thrombotic lesion (Figure 1B).The patient was treated with warfarin to target an international normalized ratio (INR) of 2.0-3. New biological assessments performed three months after the initial episode, were without any particularity (protein S at 103%). The anticoagulation was stopped, the patient continued under Aspirin 160mg, Carvedelol 6.25 mg ½ x 2/d, Ramipril 2.5 mg/d.

**Table 1:** The results of thrombophilia assessments requested

	Result	Reference value
Anticorps-antiADN	< 5 UI OMS	Négatif
Anti-anticardiolipine(IgG)	< 20 GPL-U/ml	négatif
Antithrombin activated	98%	73-129
Protein C activated	152%	70-140
Protein S activated Protein S free antigen	< 10% 105%	55-135% 55-124
Homocysteine-plasma	0.46 μmol/l 0.87 mg/l	< 13.50 < 1.82
V-proaccelereline factor	135%	62-139

## Discussion

Myocardial infarction complicating a pregnancy is a rare event, with a frequency between 3 and 7 of cases per 100,000 deliveries. Mortality associated with myocardial infarction is estimated to be between 5 and 7% [1, 3]. Since the year of 2000, the frequency of myocardial infarction has increased by pregnant women. This could be explained by the aging of the obstetrical population, by the recrudescence of cardiovascular risk factor and the easier diagnosis of myocardial infarction clinically unclear by the standardized serum troponin. The underlying triggers of acute myocardial infarction in pregnancy are considered multifactorial and attributable to physiological events occurring during pregnancy. Increased myocardial oxygen demand due to marked increases in blood volume, systolic volume and heart rate, profound changes in coagulation and fibrinolytic system leading to increased risk of thrombosis, physiological anemia and decreased diastolic blood pressure [4]. The constitutional deficits in antithrombin III (AT), protein S (PS), and protein C (PC) result in a predominant activation of coagulation which promotes the appearance of arterial and/or venous thrombosis. Deficits in PS and PC most often result in deep vein thrombosis of the lower limbs complicated or not with pulmonary embolism and cerebral thrombophlebitis, arterial thrombosis being more rare [5, 6]. Some authors explain the association of PS deficiency with arterial thrombosis by the fact that the thrombosis resulting from an endothelial lesion is favored by a pre-existing local deficit in PS. The pathogenic mechanisms of PS deficiency that lead to in vivo injury and vessel thrombosis are unknown [6]. The frequency of thrombosis is, respectively, for protein C and S deficiency, respectively, of 3-10% and 0-6% during pregnancy; 7-19% and 7-22% in the immediate postpartum [3, 5].

The morphological appearance of coronary arteries following myocardial infarction by pregnant women has been studied by angiography or autopsy mainly in the study published by Roth and El Kayam [3]. The main coronary territory reached is the former territory (70%). Before 1995, the major cause of myocardial infarction was stenosis (43% of cases), the other causes were either thrombosis (21% of cases), coronary dissection (16% of cases) and in 30% of cases coronaries were normal [4]. The atypical symptomatology of myocardial ischemia during pregnancy and the low degree of suspicion in young patients make the diagnosis less clear. The symptomatology of myocardial infraction (MI) is most often confused with the sympatic signs of pregnancy: a wave of chest pain, digestive pain suggestive of gastro-esophageal reflux [2]. The most important points in the management of patients with acute myocardial infarction during pregnancy are the timing of delivery and drug therapy. That why the medical care requires cooperation between the obstetrician, the cardiologist and the peri-perfume anesthetist in order to define the mode of delivery [7]. The management of acute myocardial infarction in pregnant patient presenting with ST segment elevation consists of medical treatment (thrombolysis) and invasive strategies. Pregnancy is not a contraindication to thrombolysis [7]. Intra-coronary thrombolysis is preferred for intravenous thrombolysis (greater potential for hemorrhagic risk, risk of abortion and premature labor). The choice of the myocardial reperfusion procedure should take into account the gestational age of the patient. In the second and third trimester, an angiographic procedure will be preferable to thrombolysis to limit the risk of maternal hemorrhage [8]. Depending on the coronary anatomy of the patient and the response to medical care, therapeutic options range from a conservative approach to percutaneous coronary intervention or coronary bypass surgery. Because percutaneous and surgical revascularizations can be very difficult in pregnancy, medical care should be considered as the first-line treatment in the absence of ongoing ischemia. It is better to avoid stent implantation and instead prefer simple balloon angioplasty for those patients because of the detrimental effects of stopping clopidogrel for abortion or delivery [9].

Pregnancy raises the question of whether or not to pursue certain drugs. Aspirin, beta-blockers with a short half-life is recommended and nitrates can be continued throughout pregnancy, without any fetal consequence. For some other drugs as clopidogrel and statins, the continuation of treatment is not codified. Several clinical cases report the administration of clopidogrel throughout pregnancy without any fetal consequence. For statins, a few cases report their use, but caution urges to use them only when there is a very pronounced familial hypercholesterolemia [10]. The date of delivery depends on the maternal state and the term of the pregnancy at the time of the infarction. In general, we try to wait for fetal lung maturation (34 weeks). Final hospitalization for monitoring and evaluation of cardiac function before delivery is required at 35 weeks. After myocardial infarction, we have to wait two weeks [9]. The delivery route is controversial. The study of maternal mortality in patients with infarction during pregnancy argues for vaginal delivery. Two studies found 23% of deaths after caesarean versus 14% after vaginal. Obstetric and cardiac conditions will determine the mode of delivery. In both cases, it should be done under intensive monitoring with epidural to reduce labor pains and heart stress. In the case of vaginal delivery, instrumental extraction is recommended in order to limit expulsive maternal efforts which increase myocardial oxygen consumption [9, 10]. The question of long-term anticoagulation after a first arterial thrombotic episode in subjects with a known protein C, S or AT deficiency remains. In the absence of prospective studies, the benefits and risks of such therapy should be assessed individually, depending on the presence of other hereditary or acquired pro-thrombotic factors, the type and degree of the deficit, the recurrence of the event and family history. Patient follow-up is therefore essential in order to evaluate the long-term impact of these abnormalities and to establish, by remote biological control, the true deficits of the transitional deficits, in order to institute possible measures therapeutic [5, 6].

## Conclusion

Myocardial infarction is a pathology that is certainly rare in pregnant women but is associated with severe maternal and fetal morbidity and mortality. Immediate and adequate management is necessary to limit the complications that can condemn the vital, functional and/or obstetrical future of these patients. The discovery of a protein S deficiency therefore requires regular monitoring to discuss a secondary prevention treatment if the deficit is confirmed. The management of the patient and her pregnancy involves a close collaboration between obstetricians, cardiologists, pediatricians and anesthesiologists to ensure optimal care.

## Competing interests

The authors declare no competing interests.
